# Mesenchymal Stromal Cells Improve Islet β-Cell Functional Survival: Analysis of Extracellular Vesicle-Trafficked Proteins and miRNAs

**DOI:** 10.3390/cells15110992

**Published:** 2026-05-28

**Authors:** Tzu-Wen Hong, Rosie Sullivan, Ryea Arora, Adya Lonsane, Zekun Lyu, Sara Caxaria, Tien-Chi Huang, Lydia F. Daniels Gatward, Thomas Burgoyne, Aileen J. F. King, Shanta J. Persaud, Peter M. Jones

**Affiliations:** 1 Department of Diabetes, School of Cardiovascular and Metabolic Medicine & Sciences, King’s College London, London SE1 1UL, UK; rosemary.sullivan@kcl.ac.uk (R.S.); ryea.1.arora@kcl.ac.uk (R.A.); adya.r.lonsane@kcl.ac.uk (A.L.); zekun.1.lyu@kcl.ac.uk (Z.L.); lydia.1.daniels_gatward@kcl.ac.uk (L.F.D.G.); aileen.king@kcl.ac.uk (A.J.F.K.); shanta.persaud@kcl.ac.uk (S.J.P.); 2 William Harvey Research Institute, Barts and the London School of Medicine and Dentistry, Queen Mary University of London, London EC1M 6BQ, UK; s.caxaria@qmul.ac.uk; 3 Institute of Clinical Sciences, Faculty of Medicine, Imperial College London, London W12 0HS, UK; t.huang12@lms.mrc.ac.uk; 4 UCL Institute of Ophthalmology, University College London, London EC1V 9EL, UK; t.burgoyne@ucl.ac.uk

**Keywords:** islet, mesenchymal stromal cells, extracellular vesicles, proteomics, microRNAs

## Abstract

Type 1 diabetes is caused by autoimmune destruction of insulin-secreting β-cells within islets of Langerhans. Transplantation of donor islets can improve glycaemic control, but current clinical islet transplantation protocols are compromised by extensive loss of β-cell functional mass soon after implantation. Co-incubation in vitro or co-transplantation in vivo of mesenchymal stromal cells (MSCs) with isolated islets improves their functional survival, although the underlying mechanisms remain obscure. Here, we show that MSC-derived extracellular vesicles (MSC-EVs) are alone sufficient to recapitulate many of the beneficial effects of MSCs on islet functional survival, offering the possibility of simple cell-free treatments to improve the outcomes of islet transplantation. We used LC- analysis and small RNA sequencing to analyse the protein and microRNA (miRNA) molecular cargos of MSC-EVs. Proteomic analysis identified >100 proteins from the Uniprot Mouse Database, including β-cell G protein-coupled receptor (GPCR) agonists which we have previously shown to enhance β-cell functional survival. MSC-EVs contained ~300 distinct miRNAs and we identified five highly enriched miRNAs that were significantly upregulated in MSC-EV-treated islets, notably miR-21a-5p. MSC-EV treatment also altered the expression of a distinct set of islet mRNAs known to be involved in islet metabolism and function. These observations may enable the further simplification of the islet pretreatment strategy by focusing on defined GMP-grade biologically active molecules rather than whole heterogeneous EV populations.

## 1. Introduction

Islet transplantation has the potential to cure type 1 diabetes (T1D) and the clinical outcomes for islet graft recipients have been improving year-on-year. However, the function of isolated human islets is severely compromised by the islet isolation process, and transplanted islets are further compromised by the hypoxic, inflammatory host environment, with consequent deleterious effects on graft survival and clinical outcomes [1,2]. Improving islet survival and function pre- and post-transplantation will improve clinical outcomes for graft recipients. Mesenchymal stromal cells (MSCs) have regenerative and supportive roles in many different tissues and pathologies [3], and our previous experimental studies have demonstrated that co-transplanting MSCs with mouse islets improved graft functional survival [4,5]. However, the clinical co-transplantation of MSCs with islets is not currently feasible because of problems associated with localising MSCs to islets after intraportal delivery. In addition, it is undesirable because of the regulatory and clinical hurdles, such as immune load, associated with transplanting additional and different cells along with the human islets. We have therefore been focusing on understanding the mechanism(s) through which MSCs influence islet function with the aim of using this knowledge to enable scalable, cell-free pre-transplantation treatment of islet graft material to improve the functional survival of human islet grafts.

Our recent work has shown that a simple pretreatment of mouse or human islets with a defined cocktail of MSC-secreted bioactive peptides protected them against cytokine-induced apoptosis and enhanced insulin secretion in vitro and this approach had beneficial effects on glycaemic control in a mouse model of diabetes in vivo [4,5,6]. However, cocktails of MSC-derived molecules have so far failed to recapitulate entirely the effects of MSC-coculture or pretreatment on β-cell function, suggesting the existence of other mechanisms. Membrane-bound extracellular vesicles (EVs) are an alternative mechanism for cell–cell communication by delivering a variety of biologically active cargos between cells, and a recent publication has implicated MSC-derived EVs (MSC-EVs) as an important route of communication between MSCs and islet cells [7].

EVs are a heterogeneous population of secreted vesicles with molecular cargos that include biologically active peptides and proteins, and microRNAs (miRNAs) [8,9]: small non-coding RNA molecules that regulate the transcription and translation of specific gene networks and which have been implicated in β-cell function. In this study, we investigated the active cargo components of MSC-EVs, with a focus on peptides/proteins and miRNAs that may be involved in the regulation of islet function. Identification of novel molecules that recapitulate the effects of MSCs and/or MSC-EVs should lead to fully defined, simple pretreatment of islet grafts without the requirement for MSCs or MSC-EVs.

## 2. Materials and Methods

### 2.1. Materials

Culture medium (DMEM and supplements), LIVE/DEAD viability assay and Total Exosome isolation kits were supplied by Thermo Fisher Scientific (Loughborough, UK) RPMI 1640 medium, collagenase type XI, and histopaque-1077 were supplied by Sigma Aldrich (Gillingham, UK). Matrigel was supplied by Corning (Amsterdam, The Netherlands). Mouse bone marrow-derived MSCs were supplied by Abbexa (Cambridge, UK). CD1 mice were from Charles River (Kent, UK). Interleukin 1-beta, interferon-gamma and tumour necrosis factor-alpha were from Peprotech (London, UK). Total RNA was extracted with the miRNeasy Mini Kit (Qiagen, Hilden, Germany). The Seahorse XFe96 extracellular flux reagent, XF basal medium and Seahorse XF Mito Stress Test kit were from Agilent Technologies (Santa Clara, CA, USA). The Caspase-Glo assay was supplied by Promega (Southampton, UK).

### 2.2. MSC Culture and Mouse Islet Isolation

Mouse bone marrow (bm) MSCs, which we have characterised previously [6], were maintained in culture (37 °C/5% CO_2_) in DMEM supplemented with 1% penicillin/streptomycin solution and 10% FBS. Mouse islets were isolated from 10- to 16-week-old male CD1 mice by collagenase digestion of pancreases as previously described [4]. Briefly, pancreases were perfused with 1 mg/mL collagenase in RPMI 1640 medium via injection through the bile duct, then digested at 37 °C for 10 min and islets were separated using Histopaque-1077 density gradients. Islets were washed in RPMI 1640 medium supplemented with 10% FBS and 1% penicillin/streptomycin before maintenance in culture overnight (37 °C/5% CO_2_).

### 2.3. Total EV Isolation

20 mL of conditioned medium (CM) was collected from 32 × 10^6^ mouse bone marrow mesenchymal stromal cells (bmMSCs) in 4xT75 flasks after 48 h incubation in DMEM. The medium was sequentially centrifuged for 10 min at 300× *g* then 2000× *g* to remove cellular debris. MSC-derived EVs were purified from CM using a Total Exosome isolation kit (Thermo Fisher Scientific, Loughborough, UK) according to the manufacturer’s instructions. Precipitated EV samples were centrifuged at 16,000× *g* for 1 h at 4 °C. EV pellets were resuspended in 100 µL PBS and stored at −80 °C prior to characterisation and analysis.

### 2.4. Nanoparticle Tracking Analysis (NTA)

A NanoSight LM10 Nanoparticle Characterisation System (Malvern, Worcestershire, UK) fitted with an electron multiplication charge-coupled device camera, and a 635 nm laser were used to determine the concentration and size distributions of isolated MSC-EVs. EVs derived from 32 × 10^6^ bmMSCs were diluted 1:1000 with PBS to a final volume of 1 mL and infused at room temperature on a syringe driver pump at a flow rate of 25 µL/s. The camera level was set to 7, gain to 1 with a detection threshold of 5–6. At least 3 videos were recorded for each sample using static mode. All EV samples passed the QC of >5 × 10^9^ particles/mL prior to cargo analyses.

### 2.5. Transmission Electron Microscopy

MSC-EVs (2 × 10^9^ particles/mL) in 100 µL PBS suspension were pipetted onto electron microscopy grids coated with formvar and left for 30 min at room temperature. 4% paraformaldehyde was added to the grids for 20 min before incubating with 20 mM glycine for 5 min at room temperature. The EVs were further fixed by placing in 1% glutaraldehyde in cacodylate buffer for 30 min and stained with UA-Zero for 1 min. Images were acquired using a JEOL 1400+ transmission electron microscope (Tokyo, Japan) fitted with an Advanced Microscopy Technologies (AMT) NanoSprint12 camera (Woburn, MA, USA).

### 2.6. Glucose-Stimulated Insulin Secretion

For quantification of glucose-stimulated insulin secretion, mouse islets were preincubated for 2 h in a physiological salt solution containing 2 mM glucose, 2 mM CaCl_2_, and 0.5 mg/mL BSA (Gey and Gey buffer [10]) to establish basal levels of insulin secretion. Groups of five islets were then incubated for 1 h at 37 °C in Gey and Gey buffer supplemented with either 2 or 20 mM glucose, then supernatants were retrieved for quantification of insulin secretion by radioimmunoassay, as previously described [11].

### 2.7. Islet Apoptosis and Viability

Induction of islet apoptosis by inflammatory cytokines was assessed by measuring caspase 3/7 activities using a Caspase-Glo assay, as previously described [11]. Briefly, mouse islets were pre-cultured alone or with MSC-derived EVs for 72 h. For the final 20 h of the culture period, half of the islets were exposed to 50 U/mL IL-1beta, 1000 U/mL interferon-gamma and 1000 U/mL tumour necrosis factor-alpha. Groups of five islets were hand-picked into 96-well plates before Caspase-Glo 3/7 reagent was added. After 1 h incubation at 37 °C, light emission was detected using a Turner Biosystems Veritas microplate luminometer (San Jose, CA, USA).

Islet cell viability was visualised using a LIVE/DEAD viability assay (Thermo Fisher Scientific, Loughborough, UK) according to the manufacturer’s recommendations. Briefly, isolated mouse islets were washed with PBS and incubated with staining solution containing calcein-AM and ethidium homodimer-1. Samples were incubated in the dark at room temperature for 30 min, excess dye was removed by washing with PBS and then fluorescence signals were detected using a confocal microscope (AXR with NSPARC). Viable cells were identified by intracellular green fluorescence resulting from calcein-AM cleavage by intracellular esterases, whereas dead or membrane-compromised cells exhibited red fluorescence due to ethidium homodimer-1 binding to nucleic acids.

### 2.8. Islet Mitochondrial Bioenergetics

Mouse islet oxygen consumption rate (OCR) was assessed using a Seahorse XFe96 Extracellular Flux Analyzer (Agilent Technologies, Santa Clara, CA, USA). For the assay, approximately 20–40 islets with basal OCR values ranging from 50 to 150 pmol/min were picked into Matrigel-coated Seahorse XF96 microplates (Agilent Technologies, Santa Clara, CA, USA) and maintained overnight in RPMI 1640 medium to allow attachment. Islets were then incubated for 1 h in XF basal medium containing 2 mM glucose to permit metabolic equilibration, and mitochondrial respiration was monitored by measuring OCR at 2 mM glucose and 20 mM glucose. Mitochondrial function was evaluated in the presence of 1.5 µM oligomycin, to inhibit ATP synthase, 2µM carbonyl cyanide-4-(trifluoromethoxy) phenylhydrazone (FCCP), to uncouple mitochondrial respiration, and 0.5 µM rotenone, to block complex I of the electron transport chain. All compounds were prepared in XF basal medium. The resulting Seahorse data were processed and analysed using Wave software (Agilent, version 2.6).

### 2.9. Proteomic Analysis

MSC-EVs (50 ng, n = 4) in 200 µL PBS suspension were sent for proteomic analysis by the King’s College London Proteomics Facility. Cysteine residues of proteins within the EVs were reduced with dithiothreitol and derivatised by treatment with iodoacetamide to form stable carbamidomethyl derivatives. Trypsin digestion was conducted overnight at 37 °C and chromatographic separation of 1 µg of extracted peptide samples was performed using a U3000 UHPLC NanoLC system (Thermo Fisher Scientific, Loughborough, UK). Peptides were resolved by reversed-phase chromatography on a 75 µm C18 Pepmap 50 cm column using a three-step linear gradient of 80% acetonitrile in 0.1% formic acid. The gradient was delivered to elute the peptides at a flow rate of 250 nL/min over 60 min starting at 5% B, then 7% B at 3 min and 40% B at 40 min, followed by a hydrophilic wash at 90% B (40.1–45 min) and an equilibration step at 5% B (45.1–60 min). The eluate was ionised by electrospray ionisation using an Orbitrap Eclipse Tribrid (Thermo Fisher Scientific, Loughborough, UK) operating under Xcalibur v4.7. The instrument was first programmed to acquire using an Orbitrap-Ion Trap method by defining a 3 s cycle time between a full MS scan and MS/MS fragmentation by collision-induced dissociation. Orbitrap spectra (FTMS1) were collected at a resolution of 120,000 over a scan range of *m*/*z* 350–1600 with an automatic gain control (AGC) setting of 4 × 10^5^ (100%) with a maximum injection time of 50 ms. Monoisotopic precursor ions were filtered using charge state (+2 to +7) with an intensity threshold set between 5 × 10^3^ and 1 × 10^20^ and a dynamic exclusion window of 35 s ± 10 ppm. MS2 precursor ions were isolated in the quadrupole set to a mass width filter of 1.6 *m*/*z*. Ion trap fragmentation spectra (ITMS2) were collected with an AGC target setting of 1 × 10^4^ (100%), with a maximum injection time of 35 ms and CID collision energy set at 35%.

Raw mass spectrometry data were processed into peak list files using Proteome Discoverer (Thermo Scientific; v2.5). The raw data file was processed and searched using the Sequest search algorithm [12] against entries in the Uniprot Mouse Taxonomy database. Database searching was performed at a stringency of 1% false discovery rate (FDR) including a decoy search. Post-translational modifications for carbamidomethylation (C; static) and oxidation (M; variable) were included in the database search.

### 2.10. RNA Analysis of bmMSC-EVs and Mouse Islets

Total RNA was extracted by TAmiRNA (Vienna, Austria) from groups of 5 × 10^9^ particles/mL bmMSC-EVs (in 500 µL PBS) and 100 mouse islets using the miRNeasy Mini Kit according to the manufacturer’s instructions. Total RNA eluted from miRNeasy mini columns in 30 µL nuclease-free water was stored at −80 °C. RNA quality and concentration were analysed with an Agilent Fragment Analyzer (Santa Clara, CA, USA).

### 2.11. Small RNA Sequencing Analysis

1 µL of miND spike-in standards (TAmiRNA, Vienna, Austria) was added to total RNAs isolated from EV and islet samples prior to small RNA library preparation using the RealSeq Biofluids library preparation kit (RealSeq Biosciences (Santa Cruz, CA, USA)/BioCat Cat. 600-00048-SOM, Heidelberg, Germany). Adapter-ligated libraries were amplified using barcoded Illumina reverse primers in combination with the Illumina forward primer. Library quality control was performed using a Small Fragment Kit (Agilent Technologies, Santa Clara, CA, USA). An equimolar pool consisting of all sequencing libraries was prepared and sequenced in single read mode with 100 cycles on an Illumina NextSeq 2000 P2 Flowcell (San Diego, CA, USA).

### 2.12. mRNA Sequencing Analysis

The QuantSeq 3′ mRNA-Seq V2 Library Prep Kit FWD with Unique Dual Indices (Lexogen, Vienna, Austria) was used for library preparation, with an input of 50 ng per sample. Briefly, after reverse transcription, the resulting cDNA was purified using magnetic beads, amplified with 14 PCR cycles, then purified again to remove any remaining contaminants. The quality of the prepared libraries was assessed using a High Sensitivity NGS Fragment Kit (Agilent Technologies, Santa Clara, CA, USA). Sequencing of an equimolar pool of all libraries was performed in single-read mode with 100 cycles on an Illumina NextSeq 2000 P2 Flowcell (San Diego, CA, USA).

### 2.13. Bioinformatics

#### 2.13.1. Small RNA-Sequencing (miND)

Overall quality of the next-generation sequencing (NGS) data was evaluated automatically and manually with fastQC v0.12 [13] and multiQC v1.14 [14]. Reads from all passing samples were adapter trimmed and quality filtered using cutadapt v3.3 [15] and filtered for a minimum length of 17 nt. Mapping steps were performed with bowtie v1.3.0 [16] and miRDeep2 v2.0.1.2 [17], whereas reads were mapped first against the genomic reference GRCm38.p6 provided by Ensembl [18] allowing for two mismatches and subsequently miRBase v22.1 [19], filtered for miRNAs of the reference only, allowing for one mismatch. For a general RNA composition overview, non-miRNA-mapped reads were mapped against RNAcentral v23.0 [20] and then assigned to various RNA species of interest. Statistical analysis of preprocessed NGS data was done with R v4.0 and the packages pheatmap vNA, pcaMethods v1.82 and genefilter v1.72. Differential expression analysis with edgeR v3.32 [21] used the quasi-likelihood negative binomial generalised log-linear model functions provided by the package. The independent filtering method of DESeq2 [22] was adapted for use with edgeR to remove low abundance miRNAs and thus optimise the FDR correction. Additional NGS QC and absolute quantification of miRNAs was done using miND^®^ spike-ins [23] based on a linear regression model.

#### 2.13.2. mRNA-Sequencing (meND)

For mRNA sequencing analysis reads from all passing samples were adapter trimmed and quality filtered using bbduk from the bbmap package v38.69 [24] and filtered for a minimum length of 17 nt and phred quality of 30. Alignment steps were performed with STAR v2.7 [25] using samtools v1.9 [26] for indexing, whereas reads were mapped against the genomic reference GRCm38.p6 provided by Ensembl [18]. Assignment of features to the mapped reads was done with htseq-count v0.13 [27]. Differential expression analysis with edgeR v3.40 [21] used the quasi-likelihood negative binomial generalised log-linear model functions provided by the package. The independent filtering method of DESeq2 [22] was adapted for use with edgeR to remove low abundance genes and thus optimise the FDR correction.

#### 2.13.3. miRNA-mRNA Interaction Analysis

Target genes of the most abundant EV miRNAs as well as differentially expressed miRNAs were identified using miRNAtap v1.42.0 [28]. This tool integrates five miRNA–mRNA interaction databases: DIANA [29], Miranda [30], PicTar [31], TargetScan [32] and miRDB [33]. Only target genes predicted by at least three of the five databases were retained for downstream analysis. To identify potential functional miRNA–mRNA interactions, the miRNA results were integrated with differential expression data from mRNA sequencing. The interactions were visualised as chord diagrams using the R package chorddiag v0.1.3.

### 2.14. Statistical Analysis

Statistical analysis was carried out using Student’s *t*-test to compare two groups or one way analysis of variance (ANOVA) test for multi-group comparisons, and *p* < 0.05 was considered statistically significant. All data are expressed as means ± SEM.

## 3. Results

### 3.1. Isolation and Characterisation of bmMSC-EVs

**Figure 1:**
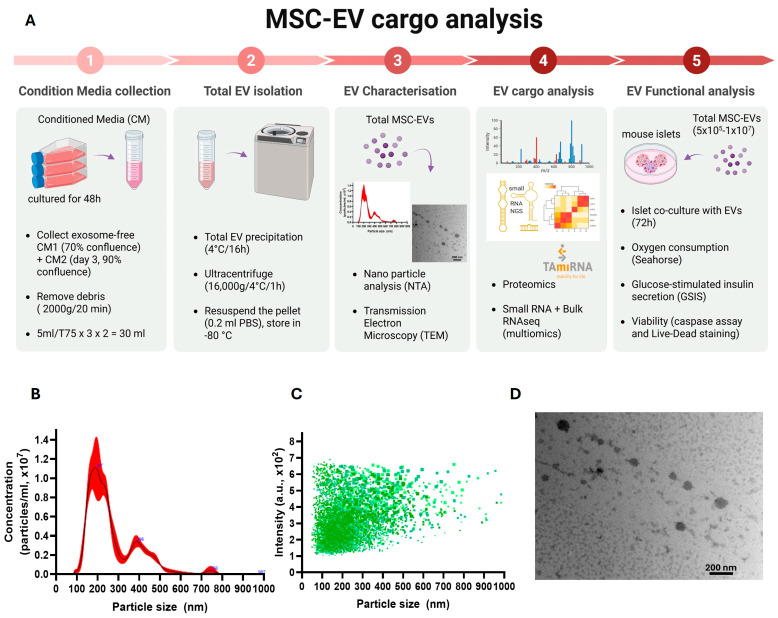
Experimental design of cargo analysis and characterisation of MSC-derived EVs. (**A**) Experimental design for the generation and characterisation of MSC-derived EVs, for cargo analysis, and for assessment of their effects on mouse islet function. (**B**) Size distribution of NTA profiles from 3 independent MSC-EV preparations showing particle concentration (particles/mL) as a function of diameter (nm) for three independent measurements on the same sample. Curves represent NTA-derived distributions. (**C**) Representative NTA scatter plot showing individual particle tracks plotted as particle size (nm) versus scattering intensity (a.u.). Each point corresponds to a single tracked particle, illustrating the heterogeneity in vesicle size and optical intensity within the sample. (**D**) Representative TEM image showing MSC-EVs with characteristic round to cup-shaped morphology and diameters consistent with nanoscale vesicles.

Total EVs were isolated from mouse bmMSCs (passages 8–14) using a total EV isolation kit (Figure 1A), then characterised with nanoparticle tracking analysis (NTA; Figure 1B,C) and transmission electron microscopy (TEM; Figure 1D). NTA revealed a heterogeneous population of particles with a predominant size distribution in the ~100–300 nm range, with a smaller proportion of larger particles extending beyond 400 nm (Figure 1B). Repeated measurements from three samples prepared from separate MSC populations showed comparable size profiles, indicating good consistency between samples. Scatter plots of particle size versus scattering intensity demonstrated a broad distribution of intensities at similar particle sizes (Figure 1C), reflecting heterogeneity in particle composition and optical properties within the sample. TEM analysis further confirmed the presence of vesicle-like structures, revealing round to cup-shaped particles with variable diameters, consistent with EV morphology (Figure 1D). Together, these data confirmed the successful isolation of a heterogeneous population of MSC-EVs.

### 3.2. Effects of MSC-EV Treatment on Mouse Islet Function

**Figure 2:**
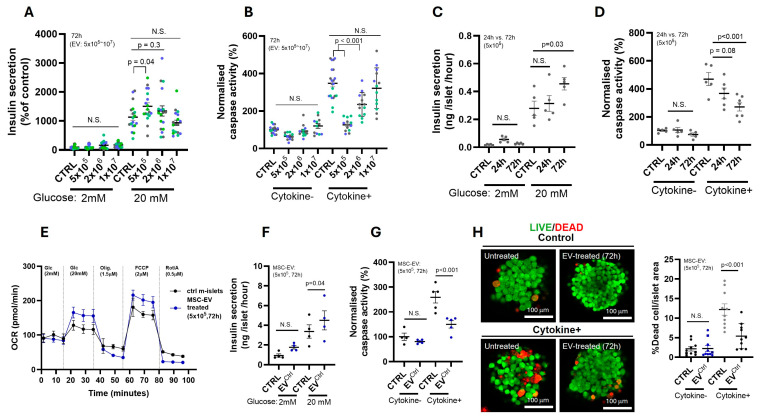
Functional effects of MSC-derived EVs on mouse islets. (**A**): MSC-EV treatment (72 h) had no effect on basal (2 mM glucose) insulin secretion. In contrast, treatment with 5 × 10^5^ particles/mL significantly enhanced 20 mM glucose-stimulated insulin secretion, an effect that was not observed with either 2 × 10^6^ or 1 × 10^7^ particles/mL. (**B**): MSC-EV treatment (5 × 10^5^ to 1 × 10^7^ particle/mL) had no effect on basal caspase activity versus control untreated islets (CTRL), but cytokine treatment significantly increased caspase activity. MSC-EV treatment (5 × 10^5^ and 2 × 10^6^ particles/mL) significantly reduced cytokine-induced caspase activity but 1 × 10^7^ particles/mL had no effect. (**C**): MSC-EV treatment (5 × 10^5^ particles/mL) had no effect on 20 mM glucose-stimulated insulin secretion after 24 h, but significantly enhanced secretion after 72 h treatment. Basal (2 mM glucose) insulin secretion was unaffected at either time point. (**D**): MSC-EV treatment (5 × 10^5^ particles/mL) had a small but not significant (*p* = 0.08) effect to reduce cytokine-induced caspase activity after 24 h, but significantly reduced caspase activity after 72 h treatment. Basal caspase activity was unaffected at either time point. (**E**): MSC-EV treatment (5 × 10^5^ particles/mL; 72 h) increased both 20 mM glucose-stimulated OCR and maximal respiratory capacity induced by 2 µM FCCP. (**F**): The batch of MSC-EVs used in (**E**) also significantly enhanced 20 mM glucose-stimulated insulin secretion from mouse islets, consistent with (**A**,**C**). (**G**): Similarly, the batch of MSC-EVs used in (**E**) significantly reduced cytokine-induced caspase activity, consistent with (**B**,**D**). (**H**): Cytokine treatment increased cell death in control islets as visualised by Live (green)/Dead (red) staining, and cell death was significantly reduced by MSC-EV treatment. Quantification of micrograph images is shown in the right-hand panel (**A**–**H**). Quantitative data are presented as mean ± SEM from independent experiments as shown in different colour, where scatter plots show individual data points representing independent biological replicates. For (**A**,**B**) N = 3; (**C**,**D**): N = 1; (**E**–**H**): N = 1 (same batch of EVs for sequencing) with technical replicates n = 4–7.

The effects of MSC-EVs on islet function were assessed in vitro. Dose-dependent experiments with three separate preparations of MSC-EVs were initially carried out to determine the most appropriate MSC-EV concentrations for subsequent experimental use. For these determinations, mouse islets were incubated with 5 × 10^5^ to 1 × 10^7^ particles/mL of MSC-EVs for 72 h after which quantifications of insulin secretion were carried out. Figure 2A shows that MSC-EVs had no effect on basal insulin secretion at 2 mM glucose. However, treatment with 5 × 10^5^ particles/mL of MSC-EVs significantly enhanced 20 mM glucose-stimulated insulin secretion, an effect that was not observed with either 2 × 10^6^ or 1 × 10^7^ particles/mL (Figure 2A). Similarly, MSC-EV treatments had no effect on basal levels of mouse islet apoptosis, but 5 × 10^5^ and 2 × 10^6^ particles/mL MSC-EV treatment significantly protected islets against cytokine-induced apoptosis (Figure 2B). In contrast, 1 × 10^7^ particles/mL of MSC-EVs did not significantly affect cytokine-stimulated mouse islet apoptosis. Furthermore, the time course experiments showed that reducing the time of exposure of islets to MSC-EVs from 72 h to 24 h did not lead to an improvement in insulin secretion (Figure 2C), nor to reduced cytokine-induced apoptosis (Figure 2D). Since treatment of islets with 5 × 10^5^ particles/mL of MSC-EVs for 72 h had consistent beneficial effects on islet functional survival (Figure 2A–D), all further functional, proteomics and miRNA analysis experiments used this pretreatment regimen (Figure 2E–H).

Insulin secretion is critically dependent on β-cell mitochondrial activity, so we assessed the effects of MSC-EVs on mouse islet mitochondrial respiration using a Seahorse XFe96 Extracellular Flux Analyzer. Exposure of mouse islets to 5 × 10^5^ particles/mL of MSC-EVs for 72 h increased both glucose-stimulated OCR (15–35 min, Figure 2E) and maximal respiratory capacity induced by FCCP (55–80 min, Figure 2E). This batch of MSC-EVs also significantly potentiated glucose-induced insulin secretion from mouse islets (Figure 2F) and reduced islet caspase activity after challenge with cytokines (Figure 2G), consistent with the data in Figure 2A–D. Visualisation of islet cell viability with LIVE/DEAD staining confirmed the protective effect of incubating islets with 5 × 10^5^ particles/mL of MSC-EVs for 72 h against cytokine-induced islet cell death (Figure 2H).

### 3.3. Proteomics Analysis of MSC-EVs

We next focused on analysing the cargos of MSC-EVs that might be responsible for these biological effects, using the same batch of MSC-EVs that had been used for the functional experiments shown in Figure 2E–H. For proteomics analysis, MSC-EVs underwent in-solution reduction, alkylation and trypsin digestion in preparation for LC-MS analysis, then label-free quantitation was performed to rank relative protein abundance. Comparison of the proteomics data against the Uniprot Mouse Database confidently identified 105 proteins within bmMSC-EVs. The top 60 most abundant proteins are shown in Table 1 ranked by their abundance, which indicates a preponderance of extracellular matrix (ECM) proteins (e.g., fibronectin, fibulins and collagen alpha chains), scaffolding proteins (e.g., filamins, myosin-9, actin, tubulin alpha chain) and ECM regulators (e.g., thrombospondins). In addition, the proteomics analysis identified that MSC-EVs contain complement 3, the parent protein of the biologically active peptide C3a, and annexin A1 (highlighted in bold in Table 1), two peptides that we have previously shown to improve islet function in vitro and in vivo [11], suggesting that MSC-EVs carry functional peptides that may contribute to the beneficial effects of MSCs on islets.

### 3.4. Differential miRNA Expression in MSC-EV-Treated Islets

**Figure 3:**
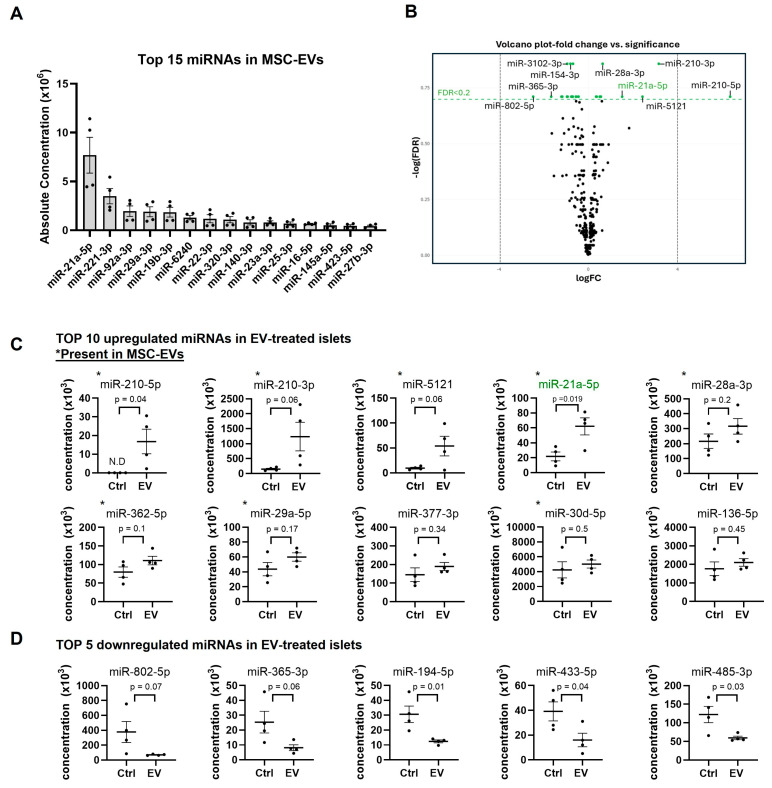
Quantification of miRNAs in MSC-EVs and MSC-EV-treated islets. (**A**): The histogram shows the 15 most abundant miRNAs detected in MSC-EVs (mean ± SEM from 4 preparations). (**B**): Volcano plot showing differential expression of miRNAs between control and EV-treated islets (5 × 10^5^ particles/mL; 72 h). Individual miRNAs shown in green indicate FDR < 0.2. (**C**): Top 10 upregulated; and (**D**): Top 5 downregulated miRNAs quantified in MSC-EV-treated islets. miRNAs are ordered by logFC (FDR < 0.2) starting with the greatest effects on the top left of each panel (mean ± SEM from 4 preparations, unpaired T-test).

Since miRNAs are one of the most common regulatory cargos trafficked by EVs [34], we also carried out small RNA sequencing in MSC-EVs, and also in islets that had been incubated for 72 h in the absence or presence of MSC-EVs (5 × 10^5^ particles/mL), to identify whether modification of islet miRNA expression in response to MSC-EV treatment could be responsible for improved islet function and survival. Analysis of the small RNA sequencing data showed distinct read composition profiles in the MSC-EVs and mouse islets (Figure S1). Thus, in the MSC-EV samples (Figure S1A) at least 60% of the reads were unmapped and the mapped reads largely corresponded to various small RNA species, the most abundant of which were rRNAs, which are essential for protein synthesis. Overall, miRNAs represented a minority population within the MSC-EV small RNA annotated reads, but it was possible to detect 322 miRNAs in MSC-EVs. The 15 most abundant miRNAs identified in MSC-EVs are shown in Figure 3A, while Figure S2A shows a broader profile that includes the 60 most highly expressed miRNAs. Expression of the most abundant MSC-EV miRNA, miR-21a-5p, was approximately two-fold that of the second highest miRNA, miR-221-3p. These data confirmed that bmMSC-EVs contain a range of quantifiable small RNAs and miRNAs.

Mouse islets also contained a range of small RNAs and, similar to MSC-EVs, rRNAs were the predominant RNA species identified (Figure S1B). There was some variability between the four samples of control islets and four samples of MSC-EV-treated islets, but the relative proportions of RNA biotypes were consistent within the experimental groups (Figure S1B), indicating reproducible library compositions. More than 500 miRNAs were detected in both control and EV-treated islets and Figure S2B,C show the 60 most highly expressed miRNAs in these groups of islets. It is clear that MSV-EVs (Figure S2A) have a different miRNA profile to mouse islets, where miR-375-3p and miR-148a-3p, two well-studied islet miRNAs [35,36], were the most abundant miRNAs in islets both before and after MSC-EV treatment. However, differential expression analysis revealed distinct changes in miRNA profiles between control islets and those that had been exposed to MSC-EVs for 72 h (Figure 3B–D). Volcano plot analysis identified multiple miRNAs that were significantly upregulated or downregulated following MSC-EV treatment based on log_2_ fold change and FDR < 0.2 (Figure 3B). The greatest increases in expression were seen in miR-210-5p, miR-210-3p, miR-5121, miR-21a-5p and miR-28a-3p (Figure 3B,C), whereas a subset of miRNAs showed significant downregulation (Figure 3B,D). The majority of the top 10 miRNAs upregulated in islets after MSC-EV treatment (Figure 3C) were also present in MSC-EVs, which might reflect transfer of miRNAs from MSC-EVs (miR-210-3p, miR-5121, miR-21a-5p, miR-28a-3p, miR-362-5p, miR-29a-5p, miR-30d-5p). Consistent with this, miR-21a-5p, the most abundant miRNA in MSC-EVs (Figure S2A), was the tenth most highly expressed miRNA in control islets (Figure S2B) but the third most abundant in MSC-EV-treated islets (Figure S2C).

### 3.5. Transcriptomic Changes and Functional Enrichment in MSC-EV-Treated Islets

**Figure 4:**
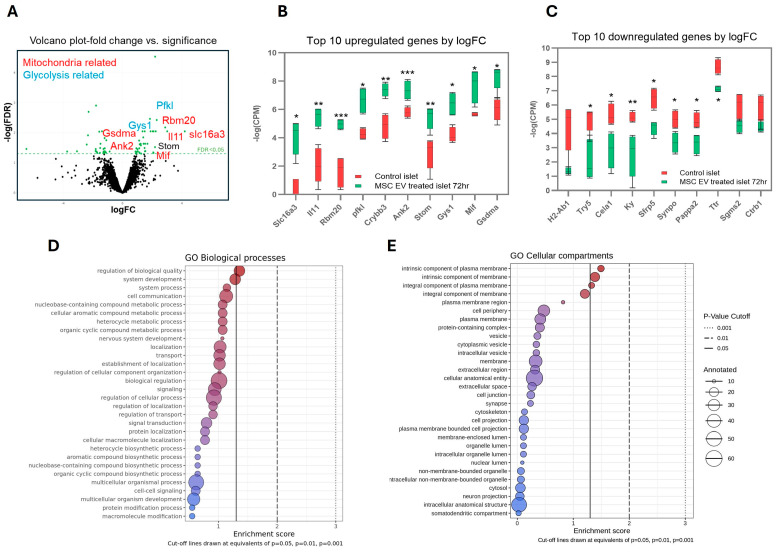

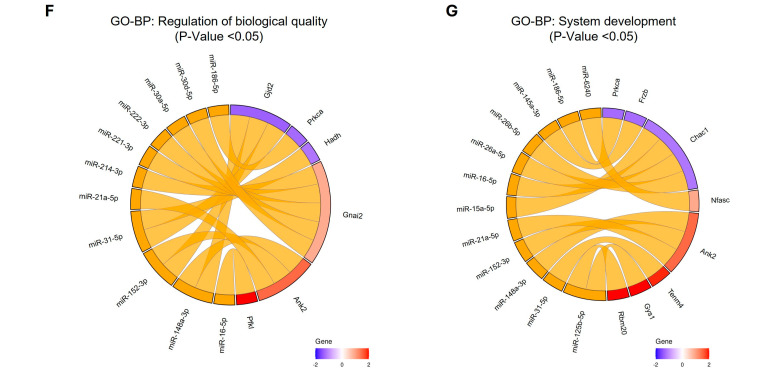
Effects of MSC-EV treatment on mRNA expression. (**A**): Volcano plot of differential expression of mRNAs between control and MSC-EV-treated islets (5 × 10^5^ particles/mL; 72 h; mean ± SEM from 4 preparations). Significantly up- or down-regulated transcripts are shown as green dots (FDR < 0.05). (**B**): Top 10 upregulated. (**C**): Top 10 downregulated mRNA species between control (red) and MSC-EV-treated (green) islets (5 × 10^5^ particles/mL, 72 h; mean ± SEM from 4 preparations. * *p* < 0.05; ** *p* < 0.01; *** *p* < 0.001, unpaired T-test). (**D**,**E**): Differentially expressed mRNAs based on an FDR < 0.05 were used in GO-term enrichment analysis. Enriched biological processes (**D**) and cellular compartments (**E**) were identified using the Kolmogorov–Smirnov test [38,39]. (**F**,**G**): Chord diagrams visualising interactions between miRNA cargo in MSC-EVs and some of their predicted mRNA targets in mouse islets reveal potential functional significance of differentially expressed mRNAs in MSC-EV-treated islets: regulation of biological quality (**F**) system development (**G**). Log2 fold change (logFC) is colour-coded according to the legend displayed (range −2 to +2).

miRNAs play key roles in regulating cellular mRNA levels, by inhibiting translation or inducing mRNA degradation [37], so we performed bulk RNAseq on both control islets and those that had been treated with MSC-EVs for 72 h to identify MSC-EV-dependent changes in islet mRNA levels. Differential gene expression analysis identified a distinct set of mRNAs that were significantly altered in MSC-EV-treated islets (Figure 4). Volcano plot analysis revealed both upregulated and downregulated transcripts based on log_2_ fold change and statistical significance of *p* < 0.05 (Figure 4A), including genes associated with glycolysis (blue text) or mitochondrial function (red text). Expression patterns of the top 10 upregulated (Figure 4B) or downregulated (Figure 4C) genes demonstrated consistent changes within the four control and MSC-EV-treated islet samples. Gene Ontology (GO) enrichment analysis of differentially expressed islet genes indicated significant enrichment of biological processes (GOBP, Figure 4D) related to cellular and metabolic regulation (regulation of biological quality), system development and process, cell communication, signalling, and response to stimulus. In addition, enriched cellular component categories (GOCC, Figure 4E) included plasma membrane, intracellular organelles and extracellular regions, suggesting broad alterations in islet cellular organisation and function following exposure to MSC-EVs. The predicted mRNA targets from the miRNAtap analysis were used in a functional pathway enrichment analysis based on an FDR cutoff < 0.05. Enriched biological processes were identified using the Kolmogorov–Smirnov test [38,39], and the enriched GOBP terms included regulation of biological quality and system development, consistent with the data generated from the bulk RNAseq analysis (Figure 4D). Chord diagrams were used to visualise the complex regulatory interactions between MSC-EV miRNAs and islet target genes involved in both regulation of biological quality (Figure 4F) and system development (Figure 4G) pathways, indicating their potential roles in post-transcriptional regulation mechanisms. Overall, these data indicated that MSC-EV treatment of islets resulted in coordinated transcriptional and functional changes. Our analysis of MSC-EV miRNA cargo and islet gene expression therefore offers a number of potential targets for future functional studies.

## 4. Discussion

EVs, particularly those derived from MSCs (MSC-EVs), have gained significant attention in recent years because of their remarkable regenerative and immunomodulatory properties [34,40]. EVs are nano-sized, membrane-bound particles that mediate intercellular communication by transferring bioactive molecules, such as proteins, lipids, and nucleic acids, to recipient cells. MSC-EVs have been shown to recapitulate many of the therapeutic effects of their parent cells, including promotion of tissue repair, angiogenesis, and modulation of inflammatory responses [41,42], while avoiding several limitations associated with cell-based therapies, such as immune rejection, tumorigenicity, and poor engraftment [43]. Due to their stability, low immunogenicity, and ability to cross biological barriers, MSC-EVs represent a promising cell-free therapeutic strategy for regenerative medicine, including improved therapies for T1D.

Our previous studies have shown that co-culture of isolated mouse or human islets with MSCs improved their functional survival by protecting them from inflammatory cytokine attack and enhancing glucose-induced insulin secretion [4,6], with consequent improvements in islet graft function in animal models of T1D [6]. In this study, we combined biophysical, ultrastructural, proteomic and transcriptomic analyses to characterise mouse bone marrow-derived MSC-EVs, and to assess their effects on gene expression and the functional survival of isolated mouse islets. In accordance with MISEV2023 recommendations for EV characterisation [44], our nanoparticle tracking analysis and transmission electron microscopy confirmed that the particles isolated from MSCs exhibited size distributions and morphologies consistent with heterogeneous EV populations, with a predominant vesicle size in the small EV range. The in vitro functional studies presented here demonstrated that treatment of isolated mouse islets with MSC-EVs was alone sufficient to recapitulate many of the beneficial effects of MSCs over a similar effective time course of 48–72 h, consistent with long-term effects via modulation of gene and/or protein expression. The effects of MSC-EV treatment on islet function were not dose-related, with lower concentrations proving more effective at enhancing glucose-induced insulin secretion and reducing cytokine-induced apoptosis than the higher concentrations used in our experiments. This may reflect potential inhibitory or non-specific toxic effects induced by the higher concentrations of MSC-EVs, so we standardised our experimental MSC-EV treatments to the lower, more effective concentration. The constraints of scalability, heterogeneity and regulatory hurdles to using MSCs to deliver therapeutic benefits to islet grafts also apply, albeit to a lesser extent, to MSC-EVs. The current study therefore aimed to identify components of the MSC-EV cargo involved in their effects on islet function to facilitate cell-free, fully defined molecular treatments of islet graft material to enhance their post-transplantation functional survival.

There are several potential mechanisms through which MSCs or MSC-derived EVs could influence islet function in vitro. We have previously used non-biassed qPCR screening to quantify G protein-coupled receptor (GPCR) expression in islets [45] and GPCR agonist expression in MSC populations [46] and demonstrated that biologically active proteins/peptides released by MSCs have beneficial effects on islet function via GPCR activation. We have further reported that a “cocktail” of these molecules enhanced insulin secretion and protected against inflammatory cytokines, and thus improved the outcomes of islet transplantation in a mouse model of T1D [11,46,47,48]. EVs are well known to transport biologically active peptides and proteins [8,9] so we here hypothesised that MSC-EVs act as a delivery system through which endogenous MSC-derived peptides and proteins influenced islet cell function in islet/MSC co-cultures. Our proteomic analysis confirmed that MSC-EVs contained all three molecules in our previous experimental “cocktail” (C3, ANXA1 and SDF1/CXCL12) with C3 and ANXA1 being in the top 60 most abundant proteins, consistent with EV-mediated delivery of these therapeutic molecules. Our proteomic analysis also highlighted the abundance of ECM-associated proteins in MSC-EVs, again consistent with our previous observations that acellularised MSC-ECM had beneficial effects on isolated mouse and human islet function [47], and with previous studies showing effects of MSC-ECM on other cell and tissue types [49,50]. Our current proteomic analysis of MSC-EVs therefore offers candidate molecules for further screening studies on islet function, and future deeper proteomic analysis of MSC-EVs may widen this potential candidate pool.

EVs are well known to traffic small RNAs, including miRNAs, between adjacent cells [34] so we also focused on the small RNA cargo molecules in our MSC-EV populations. Our sequencing analysis showed that EVs were enriched for small RNA species, in accordance with previous reports demonstrating selective loading of miRNAs into EVs [35]. Isolated islets expressed an abundance of ribosomal RNAs, as would be expected for highly active protein hormone-synthesising microorgans, in addition to a range of miRNA species. Our analysis of expression levels of miRNAs broadly agreed with previous studies. Thus, according to the online data base miRNA TissueAtlas 2025 almost 50% of miRNAs detected in our mouse bmMSC-EVs were also found in human bmMSC-EVs, and half of the most abundant miRNAs detected in our mouse bmMSC-EVs (miR-21a-5p, miR-221-3p, miR-92a-3p, miR-19b-3p, miR-320-3p) were also in the top ten most abundant miRNAs in human bmMSC-EVs, suggesting that cell-specific miRNA expression in EVs is conserved across species. Our analysis of mouse islet miRNA expression identified miR-375-3p as by far the most abundant miRNA. This miRNA was the first to be identified in mouse islets [35] where it is crucial for β-cell proliferation and function [51]. Our analysis of islet miRNA content before and after MSC-EV treatment also supported miRNA transfer from MSC-EVs to islet cells. For example, the most abundant miRNA in MSC-EVs, miR-21a-5p, was also detected in control islets but its abundance was increased by approximately five-fold after MSC-EV treatment: in islet β-cells miR-21a-5p is reported to promote glucose uptake, glucose metabolism and insulin secretion [52], and to reduce apoptosis and enhance cell survival in various tissues [53,54], making it a prime candidate for further transcriptional and functional studies in islets.

Our RNAseq analysis of the effects of MSC-EV treatment on islet mRNA expression suggests that the miRNA cargo within MSC-EVs influenced islet cell gene expression. Treatment of mouse islets with MSC-EVs induced a reproducible upregulation in a distinct set of islet mRNAs, with a concomitant downregulation of a separate set of mRNAs. A number of islet transcripts influenced by MSC-EV treatment are known to be involved in glycolysis or the regulation of mitochondrial function or metabolism, consistent with the MSC-EV-dependent changes in islet glucose metabolism, as assessed by OCR. Overall, enrichment analysis of differentially expressed mRNAs identified alterations in transcripts involved in a broad range of cellular organisation and function, which is consistent with the phenotypic changes induced in islets by prior MSC-EV treatment. Bioinformatic analysis using chord diagrams to visualise potential interactions between selected miRNA cargo molecules in MSC-EVs against their predicted mRNA targets in islets offers the further possibility of identifying key pathways through which miRNAs transferred via EVs regulate islet transcriptome expression to transduce some of the beneficial effects of MSCs on islet function.

These observations cannot alone establish causal links between specific EV-derived miRNAs and mRNA targets, but the parallel alterations in miRNA and mRNA profiles are consistent with regulatory interactions between these control mechanisms to regulate the functional responses of islet cells to MSC-EVs. Although beyond the scope of the present study, causal links between MSC-EV-induced alterations in islet miRNAs and mRNA expression can be assessed by mimicking or blocking the effects of candidate miRNAs using synthetic agomirs or antagomirs, respectively [55]. Similarly, the functional consequences of miRNA-induced modifications in islet mRNA expression can be assessed by manipulating candidate mRNA species by transient over- or under-expression using sense or antisense constructs, respectively [55,56]. Our previous studies using MSC-derived GPCR ligands demonstrated additive or synergistic effects on islet function [46,48] so it seems likely that effective treatments may require the use of several MSC-EV cargo molecules in combination for optimal effectiveness. Overall, identifying MSC-EV cargo component(s) which mimic the beneficial effects of MSC-treatment on islet functional survival [4,6] may offer a cell-free, fully defined intervention for improving the outcomes of islet transplantation as a therapy for T1D.

## 5. Conclusions

In summary, the aims of the current study were to assess the effects of MSC-EVs on islet function, and to identify MSC-EV cargo components that may be involved in changes in islet function. We have shown that MSC-EVs have beneficial effects on islet function in vitro and have identified both protein and miRNA candidates in the MSC-EV cargo with the potential to mediate these beneficial effects. These observations offer a foundation for future work to assess the potential of individual MSC-EV components to improve the functional survival of islet grafts in transplantation therapy for T1D.

## Figures and Tables

**Table 1:** Proteomic analysis of MSC-EVs. The table shows the top 60 most abundant proteins/peptides quantified in MSC-EVs, ranked top to bottom from most abundant to least abundant. Analysis is based on four separate EV preparations, and proteins highlighted in bold* have been identified previously as elements of an MSC-derived “cocktail” of molecules which enhances mouse and human islet functional survival in vitro and in vivo [9,11].

#	Description	Accession Number	Alternative ID	Molecular Weight	Abundances (Log2, %)
1	Fibronectin	P11276	Fn1	273 kDa	34.8
2	Thrombospondin-2	Q03350	Thbs2	130 kDa	31.4
3	Filamin-A	Q8BTM8	Flna	281 kDa	31.4
4	Fibulin-2	P37889	Fbln2	132 kDa	31.3
5	Collagen alpha-2(I) chain	Q01149	Col1a2	130 kDa	30.9
6	Collagen alpha-1(I) chain	P11087	Col1a1	138 kDa	30.9
7	Nucleophosmin	Q61937	Npm1	33 kDa	30.5
8	Thrombospondin-1	P35441	Thbs1	130 kDa	30.3
9	Fibulin-1	Q08879	Fbln1	78 kDa	30.2
10	Adipocyte enhancer-binding protein 1	Q640N1	Aebp1	128 kDa	29.7
11	Myosin-9	Q8VDD5	Myh9	226 kDa	29.3
12	Complement C1r-A subcomponent	Q8CG16	C1ra	80 kDa	29.3
13	Prolow-density lipoprotein receptor-related protein 1	Q91ZX7	Lrp1	505 kDa	29.3
14	T-complex protein 1 subunit gamma	P80318	Cct3	61 kDa	29.2
15	Complement C3*	**P01027**	**C3**	**186 kDa**	**28.6**
16	Pentraxin-related protein PTX3	P48759	Ptx3	42 kDa	28
17	Complement factor H	P06909	Cfh	139 kDa	27.8
18	Glyceraldehyde-3-phosphate dehydrogenase	P16858	Gapdh	36 kDa	27.8
19	Bone morphogenetic protein 1	P98063	Bmp1	112 kDa	27.8
20	Fibrillin-1	Q61554	Fbn1	312 kDa	27.6
21	Histone H4	P62806	H4c1	11 kDa	27.5
22	Procollagen C-endopeptidase enhancer 1	Q61398	Pcolce	50 kDa	27.4
23	Actin, cytoplasmic 2	P63260	Actg1	42 kDa	27.2
24	Pyruvate kinase PKM	P52480	Pkm	58 kDa	27.2
25	Collagen alpha-2(V) chain	Q3U962	Col5a2	145 kDa	27.1
26	Transitional endoplasmic reticulum ATPase	Q01853	Vcp	89 kDa	26.9
27	Actin, alpha skeletal muscle	P68134	Acta1	42 kDa	26.8
28	Collagen alpha-1(III) chain	P08121	Col3a1	139 kDa	26.7
29	Plectin	Q9QXS1	Plec	534 kDa	26.6
30	Annexin A2	P07356	Anxa2	39 kDa	26.6
31	Complement C4-B	P01029	C4b	193 kDa	26.6
32	Tenascin	Q80YX1	Tnc	232 kDa	26.6
33	Filamin-B	Q80X90	Flnb	278 kDa	26.5
34	Nidogen-2	O88322	Nid2	154 kDa	26.4
35	Collagen alpha-1(XII) chain	Q60847	Col12a1	340 kDa	26.3
36	Basement membrane-specific heparan sulfate proteoglycan core protein	Q05793	Hspg2	398 kDa	26.3
37	EGF-containing fibulin-like extracellular matrix protein 2	Q9WVJ9	Efemp2	49 kDa	26.1
38	Thrombospondin-4	Q9Z1T2	Thbs4	106 kDa	26
39	Heat shock protein HSP 90-alpha	P07901	Hsp90aa1	85 kDa	26
40	Albumin	P07724	Alb	69 kDa	25.9
41	Nidogen-1	P10493	Nid1	137 kDa	25.9
42	Elongation factor 2	P58252	Eef2	95 kDa	25.8
43	Collagen alpha-1(VI) chain	Q04857	Col6a1	108 kDa	25.8
44	Latent-transforming growth factor beta-binding protein 4	Q8K4G1	Ltbp4	179 kDa	25.7
45	Prelamin-A/C	P48678	Lmna	74 kDa	25.7
46	Heat shock protein HSP 90-beta	P11499	Hsp90ab1	83 kDa	25.6
47	Serine protease HTRA1	Q9R118	Htra1	51 kDa	25.6
48	Galectin-3-binding protein	Q07797	Lgals3bp	64 kDa	25.5
49	Tubulin alpha-1B chain	P05213	Tuba1b	50 kDa	25.4
50	Biglycan	P28653	Bgn	42 kDa	24.8
51	Complement C1s-1 subcomponent	Q8CG14	C1s1	77 kDa	24.7
52	Histone H2A type 1-K	Q8CGP7	H2ac15	14 kDa	24.7
53	Procollagen-lysine,2-oxoglutarate 5-dioxygenase 1	Q9R0E2	Plod1	84 kDa	24.6
54	Fibulin-5	Q9WVH9	Fbln5	50 kDa	24.5
55	Inter-alpha-trypsin inhibitor heavy chain H3	Q61704	Itih3	99 kDa	24.3
56	Cartilage oligomeric matrix protein	Q9R0G6	Comp	82 kDa	24.2
57	Heat shock cognate 71 kDa protein	P63017	Hspa8	71 kDa	24.2
58	Annexin A1*	**P10107**	**Anxa1**	**39 kDa**	**24**
59	EMILIN-1	Q99K41	Emilin1	108 kDa	23.8
60	Protein disulfide-isomerase A3	P27773	Pdia3	57 kDa	22.7

* Peptides with confirmed beneficial effects on islet function [9,11].

## Data Availability

The original contributions presented in this study are included in the article/Supplementary Material. Further inquiries can be directed to the corresponding author(s).

## Supplementary Materials

The following supporting information can be downloaded at: https://www.mdpi.com/article/10.3390/cells15110992/s1, Figure S1: Quality Control for quantification of miRNA content of MSC-EVs and MSC-EV-treated islets; Figure S2: Quantification of miRNA expression in MSC-EVs, control islets and MSC-EV-treated islets.
