# Supplementary material for: Mesenchymal Stromal Cells Improve Islet β-Cell Functional Survival: Analysis of Extracellular Vesicle-Trafficked Proteins and miRNAs
Source: Cells. 2026 May 28;15(11):992. doi: 10.3390/cells15110992 (PMC13256170; doi:10.3390/cells15110992)
Supplement: Supplementary file 1 [file cells-15-00992-s001.zip › cells-4296184-supplementary.pdf]

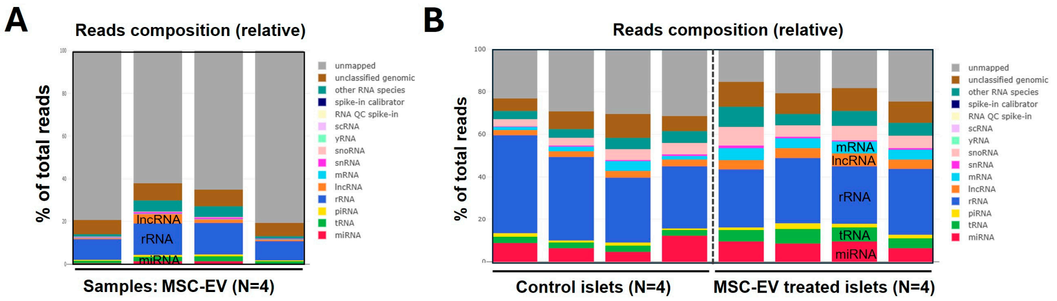

Supplementary Figure S1. Quality Control for quantification of miRNA content of MSC-EVs and MSC-EV-treated islets

Reads classification and composition showing type and origin of small RNA molecules sequences quantified in:

A. MSC-EVs (four separate preparations).

B. Control and MSC-EV-treated islets (four separate preparations).

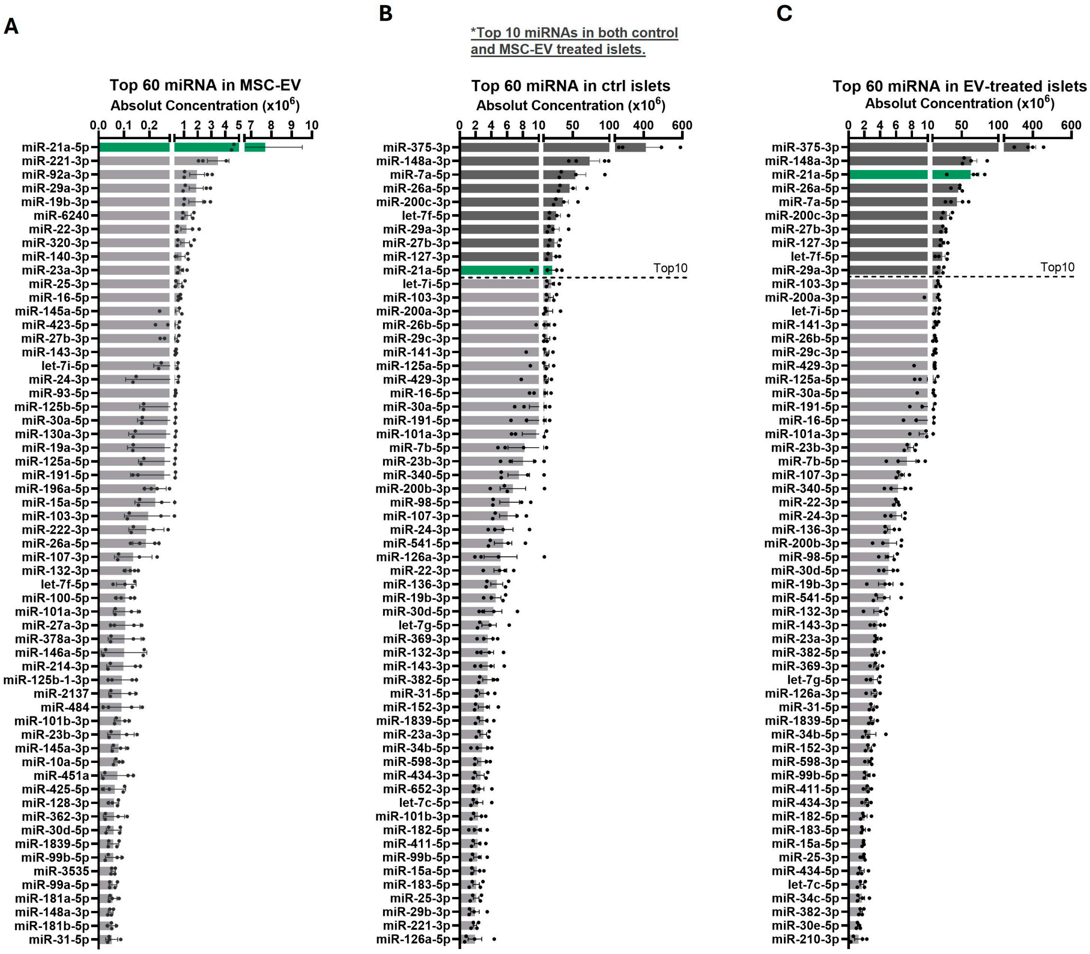

Supplementary Figure S2. Quantification of miRNA expression in MSC-EVs, control islets and MSC-EV-treated islets

The figure shows the top 60 most abundant miRNAs quantified in MSC-EVs (left panel, n=4), control mouse islets (middle panel, n=4) and MSC-EV-treated mouse islets (right panel, n=4), ranked top to bottom from most abundant to least abundant. miRNA reads were converted to absolute concentrations (molecules/uL) based on NGS spike-in calibrators. The top 10 most abundant miRNAs in control and MSC-EV-treated islets are highlighted in dark grey, while the green bars track the expression of a miRNA of potential interest, miR-21a-5p.
